# Prevention and Control of Foodborne Diseases in Middle-East North African Countries: Review of National Control Systems

**DOI:** 10.3390/ijerph17010070

**Published:** 2019-12-20

**Authors:** Dima Faour-Klingbeil, Ewen C. D. Todd

**Affiliations:** 1 School of Biological and Marine Sciences, University of Plymouth, Drake Circus, Plymouth, Devon PL4 8AA, UK; 2 Ewen Todd Consulting, Okemos, MI 48864, USA; todde@msu.edu

**Keywords:** MENA region, national food control system, food safety, food law, food inspection, risk assessment, foodborne diseases

## Abstract

Foodborne diseases continue to be a global public health problem with an estimated 600 million people falling ill annually. In return, international standards are becoming stricter which poses challenges to food trade. In light of the increasing burden of foodborne diseases, many countries in the Middle East and North Africa (MENA) region have upgraded their food laws and undertaken changes to the organizational structure of their regulatory institutions to maintain or expand international export activities, tighten control on local and imported products, and protect consumers’ health. However, until this date, the published information on the regional health burdens of foodborne diseases is very limited and it is not clear whether the recent changes will serve towards science-based and effective preventive functions and the adoption of the risk management approach. In this review, we summarize the recent food safety issues and the national food control systems of selected countries in the region although we were challenged with the scarcity of information. To this end, we examined the national food safety systems in the context of the five essential elements of the FAO/WHO Guidelines for Strengthening National Food Control Systems. These five elements—food law and regulations; food control management; inspection services; laboratory services; food monitoring; and epidemiological data, information, education, communication, and training—constitute the building blocks of a national food control system, but could also serve as tools to assess the effectiveness of the systems.

## 1. Introduction

Foodborne diseases (FBD) are a global public health issue that has major impacts on human health, livelihoods, and health care systems, but also affecting international trade through national control strategies by the implementation of food laws and regulations. With constant changes in the global food trade dynamics, food consumption behaviors, food production environment and processes and emergence and re-emergence of foodborne pathogens and chemical contaminants entering the food chain, FBD continues to be a growing problem. An estimated 600 million people, almost 1 in 10 people in the world, fall ill annually for consuming contaminated food, with diarrheal diseases being the most common form of these illnesses [1]. 

Thirty-one foodborne hazards have been identified as major causative agents resulting in 33 million DALYs worldwide, a similar order of magnitude as the ‘big three’ infectious diseases (HIV/AIDS, malaria, and tuberculosis), but also on a par with chronic cases of illness arising from air pollution [2]. The disability-adjusted life year (DALY) is a measure of overall disease burden, expressed as the number of years lost due to ill-health, disability, or early death. Forty percent of the FBD burden falls on the children under 5 years of age, with 125,000 deaths every year. Foodborne illnesses are caused by many bacterial pathogens including *Salmonella, Campylobacter, Enterohaemorrhagic Escherichia coli* (EHEC), and *Listeria monocytogenes*, but norovirus and typhoid are responsible as well for large disease burdens [3]. The 14 global subregions, defined on the basis of child and adult mortality, had considerably different burdens of FBD, with the greatest falling on the subregions in Africa, followed by the subregions in South-East Asia and the Eastern Mediterranean subregion. One reason why some parts of the world suffer more from food and waterborne diseases is that the public health structure may be compromised, and their prevention and control strategies are less well developed. These include their regulatory standards, local enforcement, educational programs, surveillance and epidemiological information systems, applied research towards advanced technologies, in addition to adverse environmental and economic conditions [4].

According to Havelaar et al. [3], the Middle East and North Africa (MENA) region is a particular one of concern. MENA countries comprise Iraq, Iran, Syria, Lebanon, Morocco, Egypt, Tunisia, Algeria, Djibouti, Oman, Israel, Yemen, Bahrain, Jordan, Malta, Saudi Arabia, Libya, Qatar, United Arab Emirates (UAE), Palestine (Gaza), Kuwait. These countries include the WHO Eastern Mediterranean region which is classified as having the third-highest estimated burden of FBDs per population, after the African and South-East Asia regions. An estimated 100 million people living in this region fall ill with an FBD illness annually and 32 million of those affected are children under five years. Nontyphoidal *Salmonella*, *Escherichia coli* (*E. coli*), norovirus, and *Campylobacter* account for 70% of the burden of FBD in this region [2].

Countries in the MENA region share relatively similar traditions, religions, and languages. However, wide socioeconomic disparities exist among them. The demography and political systems, governance, development, and climate also vary greatly. For instance, Djibouti and Yemen are classified among the least developed by the United Nations (UN) and are among the poorest countries in the world, and currently, Yemen is experiencing an extended civil war that has largely destroyed any public health system. At the other end of the spectrum are Kuwait, Qatar, and the UAE, which are among the world’s wealthiest nations as reflected in their per capita Gross Domestic Product [5]. 

The MENA region depends to a large extent on food imports which account for 25–50% of the national consumption [6]. The Gulf Cooperation Council (GCC) countries import around 33 million tons of foods every year, which is estimated to be 90% of its needs for food and drinks [7]. These are the richest countries in the MENA region (Bahrain, Kuwait, Oman, Qatar, Saudi Arabia, and the UAE) which can pay for extensive imports through oil revenues. The figures are expected to increase with the projected growth of the population and the limited potential for land expansion and scarce availability of valuable resources such as water [8]. This reality, together with the decline in agriculture, shortage of labor, and urbanization, has contributed to bringing two main issues to the forefront of the national fora attention and their international supporters: Food security and nutrition, and food safety for controlling imported foods.

Today, food security is such a growing concern that is given a high priority in the MENA region in view of the forthcoming challenges of climate change and the continuing reduction in water resources. It has urged ongoing meetings in the region to discuss the importance of water conservation, agricultural and food production [9]. Some of the MENA countries (e.g., Lebanon, Morocco, Turkey, Syria, and Tunisia) initiated the adoption of a long-term vision in establishing agricultural development strategy [8] as the lack of water policies and weak environmental legislation are leading to groundwater pollution, which further decreases groundwater quality throughout the region. In some cases, laws do not contain specific rules on solid wastes, hazardous chemicals, etc. A disproportionately large share of available freshwater is used in irrigated agriculture, but it is accompanied by intensive use of fertilizers the excess of which contributes to water quality degradation through pollution and salinization through the invasion of aquifers by seawater [10]. In this sense, given the climate change effect on weather and precipitation patterns, the MENA region is projected to see more frequent and severe droughts [11], in addition to rising challenges in food safety due to increasing temperatures and unregulated use of wastewater for agriculture and food production [12].

Where there is epidemiological information, the incidence of FBD in the region is reportedly linked to the safety of water used during food processing and production, food handling and agricultural practices (uncontrolled use of agricultural chemicals), lack of adequate food storage infrastructure, and poor enforcement of regulatory standards [13]. Accessibility to safe water and sanitation is impeded by the constant conflicts in the region. Persistent political turmoil in the MENA region complicates chances for progress being in a country that is bordering a war crisis or a country that is taking part in seemingly everlasting conflict (e.g., Lebanon, Jordan, Palestine, Yemen, Libya, and Egypt). Eight of the nineteen MENA states are among the fifty most unstable countries globally and four (Syria, Libya, Yemen, and Iraq) are considered as “failed states” due to the constant war crisis [14]. Eventually, the sub-regional variation in prosperity and stability reflects on the food safety governance and the magnitude of challenges. High-income countries (e.g., Oman, UAE, Saudi Arabia, and Kuwait) endowed with an economic strength primarily derived from oil reserves showed remarkable progress in their national food control systems and in terms of investment in water resources and food production [13]. 

The growing population, weak agricultural production and severe constraints on arable land and water scarcity make the GCC largely dependent on food imports. This together with the rising number of FBD forced these countries to strengthen their food control management systems and to undertake considerable reform to their existing systems (this is discussed in the next sections), while others (low- and middle-income countries) lag behind due to war crisis, political upheavals, and rampant corruption (e.g., Libya, Yemen, Syria, and Lebanon). Poor government, economic regression, and corruption by officials triggered the outbreak of the “Arab spring” revolutions [15]. Uprisings are continuing today such as in Lebanon [16] with devastating impacts on the economy which in turn has set the public health system and all other issues relating to food laws and regulations to a low priority.

In this review, we summarize the recent data on FBD in the region, trends and developments in national prevention and control systems while discussing the compliance of the MENA states with the five elements of the National Food Control System (NFCS) based on the FAO/WHO guidelines [17]. These elements are being used as a roadmap to determine future needs in the context of the risk management framework for the effective prevention of FBD in selected MENA countries. 

The importance of this review lies in the fact that the MENA countries are of great importance for Europe given the geographic proximity and longstanding political and economic ties [14]. Despite the non-competitive position of the region with respect to the global food export market being primarily reliant on food imports, there is a positive correlation between migration and trade and that includes food trade from immigrants’ home countries which have been reported in several European countries [18]. However, there is limited information on the food safety aspects of food imports that occur in tandem with the influx of immigrants.

## 2. Method

The sparse information on foodborne outbreaks in the MENA region led us to include not only peer-reviewed articles, but also research reports, books, media reports and news in English and Arabic, official reports, news and fact sheets published on governmental portals (local health authorities and local ministries of each country, including ministries of health, agriculture, and consumer protection), and conference proceedings. However, we recognize the importance of relying on credible information and materials that meet scientific standards, and we excluded those containing conflicting data, poor and unclear interpretations. Key-word searches were conducted using combinations of keywords in bibliographic databases: PubMed, Sciencedirect, CAB Direct and Global Health Library (WHO), Web of Science, Scopus, and GoogleScholar were used for the literature search. The search was further developed through the snowballing technique, i.e., by checking the references of the articles yielded by the initial search.

An additional search was conducted online for general publications and published news on food safety in each of the countries. 

## 3. The Pillars of a Risk Management Approach

In the light of continuous numbers of foodborne disease outbreaks and the various opportunities for food contamination throughout the global food chain, the primary goal of national food authorities is to protect public health by adopting a preventive approach for controlling food safety risks through the selection and implementation of measures that are appropriate to the risks. The traditional food safety systems tend to be reactive to events such as outbreaks and recalls, rather than being established to identify potential problems before their occurrence. The food market is globalized and characterized by complex food supply chains, competitive trade, rapidly evolving food production technologies which can lead to risks of emerging pathogens and food hazards spread beyond local boundaries. Unfortunately, traditional food safety systems are not designed to detect and identify them, and to limit the presence of pathogens and toxins. 

For almost six decades and unlike in many MENA countries, the science-based approaches build on the basis of identifying and analyzing the likelihood of hazards being present in foods, and on determining the appropriate control and preventive measures are adopted in the developed countries to reduce foodborne illnesses.

**Figure 1:**
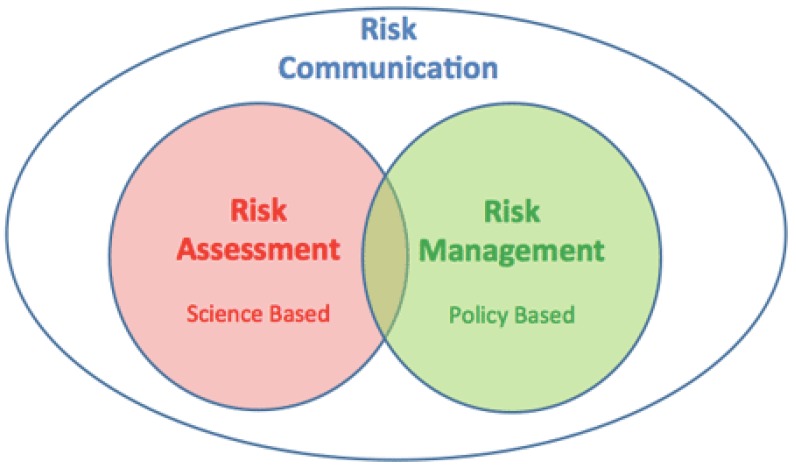
Components of the risk analysis approach. Source [21].

Science-based approaches are regarded as an integral part of risk analysis that is essential to improve food safety systems by strengthening the ability of traditional food safety systems to meet current challenges and provides a framework to collect and analyze the best available scientific information on a hazard that presents a risk to people, animals or plants in a certain country, region or even globally [19]. It is defined by Codex Alimentarius as a process consisting of three components: Risk assessment, risk management, and risk communication (Figure 1) [20]

The scope of this review does not cover these risk analysis components, rather taps on two main issues: 

The first is that the FAO report [22] has stressed that the management of food safety systems should be based on risk analysis with an integrated farm-to-table approach and it recommends the application of the Codex Alimentarius Commission (CAC) working principles [23]. This is important because the risk management has a key role in identifying food safety problems and considering the suitable policy alternatives to accept, minimize or reduce assessed risks by implementing appropriate interventions and control options. In the risk management phase, the decision-makers need to consider a range of other information in addition to the scientific risk assessment. These include, for example, most effective risk reduction actions depending on the part of the food supply chain where the problem occurs, the feasibility of controlling a risk socio-economic effects, environmental impact, a wide range of other factors legitimate to the matter under consideration [1,19]. 

The second is highlighting that risk analysis requires modern food safety and public health institutions and infrastructure, as well as an overall environment that values and supports the risk analysis paradigm [19]. Hence, the adoption of a risk management approach requires developing and improving components of food safety systems (i.e., food safety policies and food legislation, food inspection, laboratory analysis, epidemiological surveillance of food-borne diseases, monitoring systems for chemical and microbiological contamination in foods, and information, education, and communication) and is essential for an effective system capable to deal with current challenges in food safety. 

The CAC has been responsible for developing these components, standards, guidelines and other recommendations on the quality and safety of food to protect the health of consumers and to ensure fair practices in food trade. The United Nations Food and Agriculture Organization (FAO) and WHO have jointly published guidance on the strengthening of national food control systems [17] and more recently, the CAC published the “Principles and Guidelines for National Food Control Systems” [24]. These guidelines provide information for government agencies to assist in the development of national food control systems (NFCS) and to promote effective collaboration between stakeholders involved in the management and control of food safety and quality. However, different risk management decisions could be made at national levels according to different criteria and different ranges of risk management options. 

**Figure 2:**
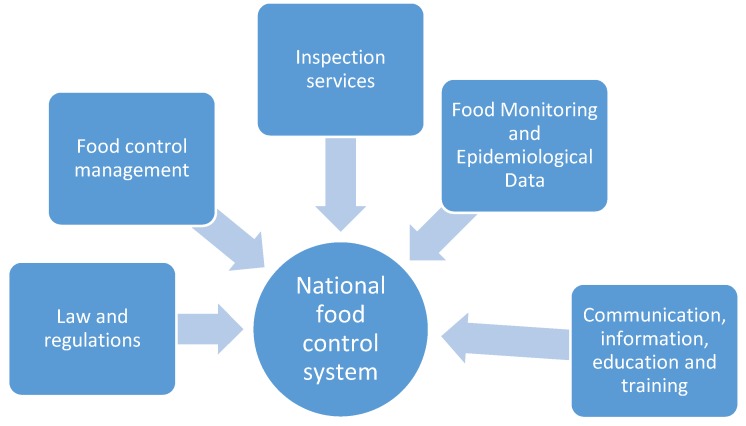
The five main elements of a National Food Safety Control System.

According to the guidelines, the five pillars of the NFCS are essential to protect the health and safety of domestic consumers (Figure 2) and to ensure there is an effective oversight system for the food supply [17]. However, the adoption of these five components is often deterred by the existence of fragmented legislation, multiple jurisdictions, and weaknesses in surveillance, monitoring, and enforcement [17]. These are common challenges in many countries in the MENA region, notwithstanding that some countries in the MENA region initiated major reforms to their food control systems. These reforms are driven to meet high national and international standards and to ensure consumer health protection in response to the rising challenges in food safety, the strict requirements for food trade, and the expanding tourism industry [25] (this is discussed in forthcoming sections). 

Nevertheless, the progress is generally not yet at the level of adopting a science-based approach except the industry has been forced to do so due to the competitive conditions for international trade. One of the widely recognized preventive approaches that are formalized by the Codex Committee is Hazard Analysis Critical Control Point (HACCP), a risk-based food safety system, that is applied in the production, processing, and handling of food products. In 1998, the European Union (EU) banned the import of fish and fisheries products from the Gulf States due to non-compliance with the EU’s environmental and health regulations based on HACCP resulting in economic losses for GCC exporters [26]. The EU ban was lifted in Oman in 1999 followed by Yemen in 2002 and the United Arab Emirates (UAE) in 2003 once quality management systems based on HACCP had been adopted [26]. The EU had made HACCP compulsory for the fisheries establishments intending to export to the EU. The recommendations of these missions prompted the amendments to regulations to ensure, for export seafood, equivalence to the EU’s standards on contaminants, additives, potable water, hygiene, and official controls [27,28].

In the next sections, we discuss current issues and perspectives in food safety and risk management in the region by examining the food safety governance in the context of the five main elements of NFCS.

## 4. Food Laws and Regulations

In the last decade, several countries in the MENA region have made substantial improvements in developing entirely new systems for the regulation and oversight of food safety, and much of these developments have been seen in the GCC countries. In recognition of the importance of food safety as a key component in agricultural development and economic sustainability, many of the systems received technical support from international organizations such as the World Health Organization (WHO) and financial assistance from organizations such as US Agency for International Development (USAID) and the World Bank [29].

The Gulf countries share similar social, political, economic, culture, religion, language, and ancestry with several similarities in their food control systems and food safety programs [25]. As the economies of these countries became interrelated and largely dependent on food imports, the GCC countries have made a major leap in the reform of their national food safety strategies taking an unprecedented interest to protect their local consumers’ health by strengthening their food control systems. One of the main achievements was the formation of the regional standardization organization, known as the Gulf Standardization Organization (GSO). GSO was established with the aim to harmonize the GCC Standards and Technical Regulations of member countries (UAE, Bahrain, Saudi Arabia, Oman, Qatar, and Kuwait) based on Codex Alimentarius and in efforts to meet the requirements the Technical Barriers to Trade (TBT) and the Sanitary and Phytosanitary (SPS) Measures Agreements [30,31].

Considerable efforts have also been directed towards the unification of the GCC countries’ food law. The GCC Committee for Food Safety (a dedicated committee of GCC Food Control Authorities) was mandated to draft a common food safety law for the region to unify the monitoring guidelines and control of imported foodstuff within the region [32]. Meanwhile, the Gulf Rapid Alert System for Food (GRASF) connecting all the members of the GCC states was established in 2012 by the GCC Food Safety Committee to provide means for rapid exchange of information on food alerts and food scares flagging implicated food products to allow prompt regulatory actions [25,33]

Similarly, Oman promulgated a new Food Safety Law in 2008 aiming at safeguarding public health and strengthening consumer safety. The new law provides a legal basis for the government’s plans to establish a food management regime. The Ministry of Regional Municipalities and Water Resources, the agency mandated for the enforcement of the new law, has outlined a national framework for ensuring the safety of food. As part of this framework, a national food and drug authority was established with the goal to raise food safety to international standards. The upgrade of the national food safety control system was specifically seen in Saudi Arabia; Saudi Arabia is one of the countries that have been able to comply with Codex Alimentarius and other standards recognized by the World Trade Organization, such as the SPS and TBT, and has adopted a strongly worded resolution that recognizes food safety as an essential public health function. 

In North Africa, Tunisia approved a new food safety law which mandates the National Agency of Sanitary and Environmental Control Products (ANCSEP) as a competent authority for food safety risk assessment including GM Food safety assessment (mentioned in the forthcoming section) [34,35]. Tunisian food legislations are currently incorporated in several general laws intended to organize the food sector and to protect the consumer (law # 92-117, 1992). According to the Tunisian Ministry of Health, the new law is foreseen to boost export opportunities and ensure the highest level of food hygiene throughout the food chain [35].

Recently Lebanon passed the food safety law with a view to food sector reform [36]. The law covers all types of food and beverages, including processed foods. It describes the regulatory requirements of food safety from farms to fork and establishes the structure of the public governance of food safety. However, the law is not yet in force, but also Lebanon is still working on the legislation required to enter the World Trade Organization [9] while facing many challenges, i.e., the conflict in Syria and the refugee crisis, in addition to political turmoil, and failure of economic growth. Egypt as well witnesses turbulent times and political conflicts yet started working towards the modernization of the food safety systems, strengthening the capacity of analytical laboratories, and establishment of a food inspection-based risk analysis by establishing the National Food Safety Authority (NFSA) (discussed in the next section) [37]. 

## 5. Food Control Management

Food control management has been defined as “the mandatory regulatory activity of the enforcement of food laws and regulations by national or local authorities to provide consumer protection and ensure that all foods during production, handling, storage, processing, and distribution are safe, wholesome and fit for human consumption; conform to safety and quality requirements, and are honestly and accurately labelled as prescribed by law” [20]. For successful management of food safety control systems, involvement and partnerships of the various sectors of the economy (producers, traders, industry and government and the contribution of the scientific community) are a pre-requisite [32]. 

National food control systems are vital tools in governing the safety and quality of food intended for human consumption. The advancement in the reform of existing food control systems and institutional frameworks in the region is quite recent. It was not until January 2017 when the Egyptian Parliament established the National Food Safety Authority (NFSA) by virtue of the new Law 1/2017 [37,38]. According to the new law, NFSA shall exclusively assume all the responsibilities and jurisdiction of all ministries, public institutions, government agencies, and municipalities in relation to supervision over the handling of foodstuff with the aim to improve the regulatory oversight and efficiency in the food system [37,39]. NFSA is the key actor in the Arab Food Safety Initiative for Trade Facilitation (SAFE), a project implemented by the United Nations Industrial Development Organization (UNIDO) with the aim to facilitate regional trade in food/agri-based products through strengthening the regional coordination and harmonization mechanisms on conformity assessment and Food Safety systems in accordance with international standards and best practices [40,41]. 

Similarly, the new federal law on food safety and the Ministerial Decree No. 14 of 2016 on controlling imported food for non-trading purposes in UAE were approved in 2016. This was followed by integrating a risk-based approach in the inspection activities, the launch of the National Rapid Alert System for Food in 2017, and the establishment of the National Food Accreditation and Registration System in 2018 that serves as an integrated smart platform for food products data [42]. The food safety control in the emirates of Dubai and Sharjah falls under the scope of the respective municipal authorities, whereas in the emirate of Abu Dhabi, Abu Dhabi Agriculture, and Food Safety Authority plays a central role to ensure food safety and guarantees that the food is fit for human consumption.

Another example is the government in Oman deciding to renew their food safety system. A study of the Omani system was conducted to evaluate the effectiveness of the current food controls in place for protecting public health from emerging biological and chemical hazards. The study survey was undertaken within the different food safety authorities in Oman to examine the different elements of the national food control systems in terms of their existing food control management, food legislation, food inspection, food analysis laboratories, and information, education, and communications. Officials from the different authorities were interviewed and results were captured in prepared questionnaires. Overall examinations of the challenges, strengths, and weaknesses of the existing system have been highlighted. The findings of the study indicate significant progress is being made and the creation by the government of a national Centre for Food Safety and Quality is a significant positive step [32]. 

Recently, many countries have undergone reorganizations for their food safety control functions, the common feature was the assignment of control and monitoring of food safety along the food chain for a single agency. Currently, besides the NFSA, the region includes the following food safety regulatory institutions: Jordanian Food and Drug Authority (JFDA); Agence Nationale de contrôle Sanitaire et Environnemental des Produits (ANCSEP, Tunis, Tunisia); Office National de Sécurité Sanitaire des Produits (ONSSA, Rabat, Morocco); Ministry of Health (Abu Dhabi); and Saudi Food Drug Authority (Saudi Arabia). 

Saudi Arabia has undergone a major transition from fragmented organizations to a centralized food safety administration, by establishing in 2003 the Saudi Food and Drug Authority (SFDA) as an independent body that directly reports to the Prime Minister and responsible to regulate, oversee, and control food, drug, medical devices, as well as set mandatory standard specifications for imported or locally manufactured products [25,43].

Despite the engagement of several MENA countries to consider a profound reform of their food safety systems, the situation is still far from being adequate since the time of the published report of FAO/WHO [44]. There are cases where laws and regulations are available, yet they are not enforced [8] and where violations and food outbreaks are handled with a punitive approach often overlooking investigation of the root causes which constitutes the basis for establishing national policies to reduce the likelihood of recurrence [45]. Food controls in the MENA countries are generally operating through a multi-agency approach, although few countries are outliers to this fact. Various ministries and municipalities in Algeria, Iraq, Egypt, Lebanon, Libya, Morocco, Syria, Oman, and Tunisia have overlapping responsibilities in food control [32,35,38,44,46,47,48]. The duplication of roles is often associated with conflicts over jurisdiction and a fragmented regulatory framework which leads to weak enforcement and inefficiency in identifying food safety issues, hence deterring any chance to advance towards effective preventive and science-based systems.

A typical example of a fragmented regulatory framework is Lebanon, previously mentioned as having passed a food safety law, but yet to be enacted. According to this new law, a Food Safety Lebanese Commission (FSLC) shall be created as the referral body to implement and oversee the regulations, and implementations [36,47,49]. However, the members of the FSLC have not been assigned to this date due to conflicts in ministerial roles and disagreements within the cabinet on developing an independent body, therefore the new law is not yet enforced [47,50]. Currently, this issue is relegated to the minimum level of priorities of local authorities amidst the growing environmental problems and pollution and resulted in poor compliance with the international food safety standards and a poor regulatory framework (e.g., certification, quarantines, pesticide applications) which hinders expanded food trade [51].

In Tunisia the food controls activities are coordinated by the National Agency of the Sanitary and Environmental Control of Products (ANCSEP) that was created in 1999 to ensure the observance of national and international standards in matters of sanitary and environmental food controls. Nonetheless, adequate food control management in Tunisia is deterred by some difficulties that were attributed by the Minister of Health to the lack of coordination between the different stakeholders, limited competent human resources and analytical capacity, and the legal and institutional framework that is scattered among several ministries. In addition, Tunisia, and that include other MENA countries, lack of a food risk assessment framework [35]. 

The approach for these countries is different from food safety control management in Kuwait, Oman, and the emirate of Dubai in UAE. These give local municipalities a central role and authority power in executing the regulations [25]. In Kuwait, the food safety responsibilities are currently shared among the municipality, Ministry of Health, the Public Authority for Agriculture Affairs and Fisheries Resources, the Public Authority for Industry, the Ministry of Commerce and the Office of Consumer Protection [46]. Likewise, the regulatory enforcement is scattered and fragmented through different organizations in Oman resulting in unharmonized inspection procedures within the regions of the country and different levels of controls applied over the food produced domestically and those for export markets [32,47]. Yet, with the Royal Decree that was issued in 2019 and approved the establishment of a national Centre for Food Safety and Quality (CFSQ) in Oman, it is anticipated that the implementation of quality and safety standards throughout the food supply chain shall be overseen under a unified system and standardized inspection procedures. The center will be equipped with laboratories to carry out all the various analyses for the protection of public health, licensing of food handlers, national capacity-building, as well as the implementation of scientific studies and research in all fields related to the safety and quality of food [32]. 

The progress is quite mixed in the MENA region given the disparities in political and socioeconomic status. For example, in Iraq, the food control at the entry points is no longer enforced due to insecure conditions and customs facilities that were damaged during the war. In January 2019, a WHO mission assessed the food safety and quality in Iraq in response to the request of the Iraqi Ministry of Health and Environment. The resulting joint workshop on food safety and quality assurance indicated several food safety issues and recommended key elements to be addressed for an effective food safety system: Intersectoral coordination and implementation of laws and regulations, Emergency preparedness and response food safety policy, monitoring and surveillance system, a food safety control and inspection system, information and communication, human and financial resources [29].

Also, in Palestine, significant operational weaknesses were recently discussed in a review and assessment of the legislation pertaining to food safety. The legal framework regulating food safety is fragmented functioning with no coordination among involved parties which leads to foodborne illnesses being unreported, improperly identified that the vast majority are attributed to unknown reasons. Not only the current food safety legislations are not harmonized with international standards, but also the absence of control over borders is leading to an immense problem of smuggled food products to the domestic market before being monitored by the Palestinian Ministries of Health and Agriculture [8].

In principle, the establishment or update of national food control systems requires national authorities to reinforce their control activities by adopting a preventive approach to mitigate the risks across the food chain, developing science-based food control strategies, establishing priorities based on risk analysis and efficacy in risk management [17]. Governments should recognize the application of the HACCP approach by the food industry as a fundamental tool for improving the safety of food [17]. Although the Best Management Practices (BMP) and quality assurance systems such as HACCP have been introduced throughout the region, they are not fully integrated into the domestic inspection systems which continue to focus primarily on end-product control. In a number of countries, many industries apply HACCP and other quality management standards on a voluntary basis in order to improve food safety domestically as well as increase their share of export markets [35,52,53].

## 6. Surveillance, Food Monitoring, and Epidemiological Data

As indicated earlier, data on FBDs in the MENA region are limited, especially relating to surveillance practices. For instance, in searching the Sciencedirect database, the search keywords surveillance + the Middle East and North African countries + food outbreaks yielded 47 articles. Similarly, foodborne illnesses + surveillance + Middle East and North African countries produced 48 articles, from those and in both searches, only two research articles originating from Oman and Saudi Arabia were included for their relevance to the region and the subject. On the other hand, 10 articles were retrieved from PubMed out of which only three were relevant to the topic, hence considered in this review.

Apruzzese [54] also reported a low number of hits when searching for keywords on food safety in developing countries, which implies that developing countries are currently facing significant challenges in detecting foodborne outbreaks and/or in reporting outbreaks in publishable articles. Consequently, when foodborne outbreaks often go unrecognized, unreported or uninvestigated, building a scientific opinion on the risk profile of many countries becomes a complex task [55].

Basically, the prevalence of FBDs in the MENA region is still generally not well understood because of the ineffective FBDs surveillance and many cases are perceived as mild and self-limiting or unverified due to gaps in detection, surveillance and reporting [32,56]. According to the FAO report and available surveillance data, the available burden of FBD varied greatly among Arab countries, including those in the MENA region (Table 1). However, some of these data are old and recent information is not available to show improvements or declines in the reporting system in all countries. For instance, the declining trend of reported outbreaks in Oman and Saudi Arabia [57] could mean fewer illnesses or less reporting of illnesses. In Lebanon, the surveillance data include reported cases related to mandatory notifiable diseases for at least the past 10 years. In other countries, some of the data were captured from research articles or published news.

For accurate data on the prevalence of FBDs, control laboratories should have both the resources, capacity, and capability to identify and detect the source of microbial hazards. This is particularly important in the light of the increasing risks and emerging threats to public health that occur in tandem with new food technologies, global food marketing and broad exposure of food products to hazards throughout the global food chain. According to literature and available information, reporting of foodborne illnesses is still ineffectively practiced and exact information on implicated foods are limited, notwithstanding few countries have the required resources to monitor and to advance the reporting of FBDs which is actually showing improvements in Jordan, Kuwait, Oman, Saudi Arabia, and UAE. 

In Oman, promising developments are underway. Most of the regulatory laboratories have adequate infrastructure and well equipped to perform analytical tests essential for an effective food control system [58]. It is assumed that the slight increase in the incidences of FBDs in Oman in 2010 was linked to the strengthening of laboratories capacities, as they remained fairly constant between 1985 and 2013, noting that the cases peaked in the summer period as temperatures can reach an average 45 °C [32]. In addition, the Central Public Health Laboratory (CPHL) in the Directorate of General of Health Affairs of the MOH in Oman was identified as the coordinating laboratory for the regional collaborative surveillance network for foodborne infections known as the PulseNet Middle East. PulseNet Middle East is a network that is part of the PulseNet International network and was established by WHO, CDC, and the Naval Medical Research Unit Three (NAMRU-3) in December 2006 for molecular surveillance of foodborne infectious diseases to support the regional food safety plan and promote technical collaboration among countries. It includes public health laboratories and academic and medical institutions from 10 countries in the WHO Eastern Mediterranean Region (Bahrain, Iran, Jordan, Kuwait, Lebanon, Libya, Oman, Palestinian Authority, Qatar, Saudi Arabia). Participants perform pulsed-field gel electrophoresis (PFGE) on disease-causing bacteria isolated from patients by following standardized procedures strictly developed by CDC [59].

PulseNet Lebanon was established in 2011 through the collaboration between the Epidemiological Surveillance Program (ESPMOH) at the Ministry of Public Health (MoPH), the Faculty of Medicine at the American University of Beirut (AUB), and the Lebanese Agricultural Research Institute (LARI) of the Ministry of Agriculture, with the support of WHO and US Centers for Disease Control and Prevention (CDC). Through this network, performing DNA fingerprinting patterns by pulsed field gel electrophoresis (PFGE) enabled the link between human foodborne illness and food source of infection in outbreaks. A total of 1747 cases including four deaths were reported to the Ministry of Public health between 2009 and 2013. Most reported suspected food products were ground raw meat (25%) and cooked chicken (11%) which match well with the Lebanese dietary habits that include raw meat [60]. 

During this period, in a separate study, 290 clinical and 49 food isolates were identified as *Salmonella* [60]. Serotyping revealed the prevalence of ten and seven serotypes in the clinical and food samples, respectively. Fifty-one isolates from chicken ceca and carcass were identified to be *Campylobacter* spp. Fifty-nine samples were identified to be *Listeria monocytogenes*. The common serotypes isolated in both clinical and food samples were: *Salmonella* Typhimurium, *Salmonella* Enteritidis, *Salmonella* Braenderup, *Salmonella* Typhi, *Salmonella* Paratyphi A, *Salmonella* Blockley, and *Salmonella* Newport [60]. The same study showed that the 2011 *Salmonella* Typhi outbreak in Nabatiyeh (south of Lebanon) was traced to raw meat and Enteritidis in Mount Lebanon was traced to Arabic sweets, while the 2009 incidence of salmonellosis was associated with the consumption of undercooked chicken products at a bank cafeteria [61]. It is reported that 48% of shawarma sandwiches in Lebanon were contaminated with *Salmonella* Paratyphi [62].

There are no published data on the FBD of other PulseNet member countries. That is probably due to the fact that the certification of laboratories has lapsed because of inactivity over a two-year period, and the membership criteria and procedures had never been formalized, resulting in some member countries being dropped from PulseNet [59]. Nonetheless, NAMRU-3 has provided training for laboratorians in Jordan, Palestine, Lebanon, Egypt, Morocco, Libya, Kuwait, and Iran since 2007, and recently in 2014 for Iraqi scientists (although Iraq is still not a member of this network) on the use of PulseNet, but they were still expected to get the correct equipment before they would be able to implement the program [63]. 

Similarly, the improvement in laboratory capacities in Saudi Arabia reflected an increasing number of food poisoning cases that are attributed to norovirus [43]. In 2006, 31 foodborne illness outbreaks were attributed to *Salmonella* spp., followed by *Staphylococcus aureus* and often related to the consumption of Mediterranean meat sandwiches [64]. *Salmonella*, *Staphylococcus aureus*, *E. coli*, *Bacillus cereus*, and *Shigella* were major causative agents linked to 16 reported in earlier outbreaks in 2003 [57] and *staphylococcus aureus* was associated with 41% of bacterial food poisoning cases [65].

In general, gastrointestinal infections are frequent in this region primarily caused by *Salmonella* spp., followed by *Shigella* spp., and other pathogens including hepatitis A virus and parasites. Unpasteurized dairy products were implicated in FBDs reported in Algeria, Jordan, Kuwait, Oman, Saudi Arabia, Lebanon, the Palestinian Authority, and Syria [57,71,72]. Recent data from Oman showed that *Salmonella* was the most common cause of FBD, often linked to the consumption of commercial meat products [32,64]. 

To prevent foodborne illnesses, the Dubai municipality’s food safety department made a significant improvement to the strategic control plans by building a comprehensive database and statistics on foodborne illnesses that will help to develop food legislation in collaboration with the US Centers for Disease Control and Prevention (CDC) [45]. With a specialized team that includes trained experts from the CDC and WHO, 57 hospitals and clinics were trained on the procedures of monitoring and investigating disease transmission [73]. Data from the first foodborne disease investigation and surveillance system in Dubai revealed 1663 cases reported in the first nine months of 2011 while the number of confirmed cases was not still declared at that point in time [74]. Similarly, in 2013, over 1120 cases of suspected FBD were reported, half of them (550 cases) was not confirmed [56]. Among the verified cases, 214 and 137 cases of amoebic dysentery and typhoid/paratyphoid were confirmed, respectively and 43 cases of hepatitis A. Twenty and 10 cases of giardiasis and shigellosis were reported, respectively. *E. coli* and *Campylobacter* species were confirmed each in one case, and 3 cases were related to *Campylobacter jejuni*, and five cases to bacillary dysentery (likely unconfirmed *Shigella* infections) [68].

The climate in most if not all of the MENA countries are characterized by high summer temperatures, and the GCC countries have longer periods of hot days. Therefore, it is expected to observe higher cases of food poisoning during the very warm seasons. Inadequate time intervals/storage temperature constitutes a common risk factor for food poisoning and is reportedly behind more than 800 cases of food poisoning in Dubai during the first half of 2018 with 200 cases being caused by *Salmonella* and the rest remained unverified [69] and 64.5% of food outbreaks reported in Saudi Arabia in 2006 [64].

Data from Cairo, Egypt, and Morocco are sparse. In the latter, the consumption of sausage products contaminated with *C*. botulinum resulted in 78 cases and 20 deaths [75]. In 2017, a food outbreak occurred in Meknes at a familial dinner, 9 individuals were hospitalized. Clinical signs suggested the implication of *Staphylococcus* aureus [76]. In Cairo, the outbreak of foodborne botulism in 1991 was associated with the consumption of faseikh (a commercial raw salt fish) resulting in 91 cases and 18 deaths [77]. In 2013, 160 university students were reported ill after eating tuna-containing meals on campus. This was followed two years later *by hundreds of students in the same university who fell ill after eating food contaminated with Salmonella* [78]. In 2017, hundreds of schoolchildren were admitted to hospital in a suspected mass food poisoning. 

Recently, the Minister of Health in Tunisia revealed that the number of cases of food poisoning increased from 1015 cases in 2017 to 1855 cases in 2018 [35]. These reports or other news published information rarely identify the causes of foodborne illnesses. For instance, incidences of tourists contracting FBDs when visited resorts in Turkey and Egypt were usually of unknown origin but eventually linked to poor hygienic conditions and often solved by filing lawsuits against the resorts [4]. 

Closure of food facilities following publicly reported food poisoning, intentional adulterations, and violations come often with no further and exact investigation of the source of causative agents to devise adequate risk management strategies. Unawareness and underestimation of food safety risks by policy decision-makers hinder progression in risk management in the region. The Director of the Department of Communicable Diseases in Jordan stated that the poisoning cases in the Kingdom are “normal” and caused by improper handling of food or washing of vegetables and fruits or for lack of personal hygiene [67]. According to the Ministry of Health’s epidemiological monitoring report of the Directorate of Communicable Diseases in Jordan, the Ministry of Health receives daily reports of food poisoning, but few have been confirmed, and the reported cases are significantly higher than those reported by the epidemiological department of the Directorate of Communicable Diseases. Thirteen incidents have been recorded since the beginning of 2014 till October of that same year with 250 people fell ill. In the northern Jordan Valley, a food outbreak led to 133 hospitalized and was attributed to “eating spoiled foods”. The earlier year also witnessed the high number of food poisoning with a total of 247 reported cases in different regions followed by shigellosis affecting 145 people and salmonellosis in norther Jordan Valley affecting 147 people. 

In 2011, 410 cases of food poisoning in Tunisia were attributed to *Salmonella* spp. Yet, a senior officer responsible in the Ministry of Health considered the pathogen was not dangerous, and the health impact would be limited [79]. Later, in response to a nation-wide scandals of expired meats and food products in the market and illegal use of raw sewage in fresh produce irrigation, it was ascertained that the rising number of food poisoning cases among university and school children resulted from consuming spoiled food products, with no reference to causative agents, but possible implication of mishandling practices during preparation or processing. The member of the Committee on Agriculture (Agriculture), food security, trade and services in the Tunisian Parliament indicated that the suitable approach that is being discussed in the parliament is strengthening penalties up to 7 years imprisonment and fines of about $70,000 USD in case of food safety violations and creating a unified legal framework [80].

These gaps in information call for concerted efforts to enhance the surveillance system in the region [32,81] mainly in countries where available laboratory analytical support for public health agencies is often minimal or lacking, even though some research institutions may have up-to-date equipment and technical expertise [4]. Addressing other challenges resulting from the lack of harmonization of methods and techniques, non-existing coordination among the different laboratories, and limited training on advanced methodologies and techniques are key for improving the surveillance mechanisms [25]. 

There are continuous efforts for strengthening the capacity of analytical laboratories and the enhancement of their infrastructure [32,35,40,82], albeit rather slowly and the realizations and achievements in capacity building are seemingly possible with supports of international bodies [59,82]. The poor performance for prevention and control of FBD and slow progress towards the five main elements of a National Food Safety Control System for most of the MENA countries may be ascribed to a lack of commitment to advance and further the surveillance program through improved communication, particularly risk communication and conducting periodical PulseNet training courses in the Region tailored to the level of PulseNet laboratory capacities of the participating countries [59]. 

Nevertheless, with the mission to establish a regional, laboratory-based surveillance network on foodborne diseases, the Middle East Consortium on Infectious Disease Surveillance (MECIDS) was formed in 2002 with representative members of the ministries of health and academia in Jordan, the Palestinian Authority, and Israel. The main function of MECIDS was to develop harmonized methodologies and laboratory capacities and to establish a common platform for communication, data sharing and analysis, and coordination of intervention strategies. This was considered a major step towards assessing the burden of foodborne diseases in the region with data collection starting in 2005 and the surveillance of salmonellosis being the first to target. The microbiological laboratories were selected to form the network of sentinel laboratories covering the different districts of each country and one laboratory was designated as the National Reference Laboratory (NRL). Data analysis units have been established to manage the data and serve as a central point of contact in each country, while the regional data analysis unit, the Cooperative Monitoring Centre (CMC) is located in Amman, Jordan and assigned as the regional unit for sharing data from the national systems. MECIDS has also served in capacity building of its members through joint training courses on interventional epidemiology and laboratory technologies [83]. 

Capacity building of surveillance laboratorians is important in the region to keep up with the fast-paced advancement in analytical methods, such as the whole-genome sequencing (WGS). WGS is already a routine tool used in epidemiological investigations of foodborne outbreaks to identify and characterize pathogens in developed countries [84]. It is considered a novel analytical technology that brings major public health benefits by minimizing the scale of the outbreaks and preventing recurrence of the problem from the same source [85], and for its suitability to identify virulence, antimicrobial-resistant strains and other emerging new foodborne pathogen strains [86]. According to Apruzzesse [54], while incorporation of WGS into regulatory frameworks in developed countries has demonstrated its effectiveness in food safety management, there was no data that confirm or indicate the incorporation of the WGS into the national food control systems in any developing country which was further confirmed by the focus group session with experts from developing countries. The focus group included only Ghana, Iran, the Philippines, Sudan, Tanzania, and Thailand and the limitations were mainly related to IT-related issues, lack of knowledge and vision at governmental levels. 

We may only assume a comparable situation in some MENA countries given the lack of published data and studies. The 2018 Workshop on the coordination and capacity-building of the PulseNet Middle East laboratory network reviewed the current status of foodborne disease surveillance activities of the member countries. Countries were classified into three tiers based on their level of implementation and related needs. Tier 1 (Iraq, Libya, Pakistan, and Sudan) comprises countries that are just starting molecular surveillance and therefore need either PFGE equipment or BioNumerics software, or both, along with personnel training on how to perform PFGE and analyze the results. Tier 2 (Egypt, Islamic Republic of Iran, Jordan, Kuwait, Morocco, Palestine, and United Arab Emirates) consists of countries that have all necessary equipment and software, and experience with PFGE, but no plans yet of switching to WGS (even though the Dubai municipal laboratory in the UAE has capacity for WGS); these countries are most in need of training on BioNumerics software and certification. MENA counties need to consider WGS to be able to monitor and report on FBD surveillance leading to better prevention and control strategies. And in fact, the countries of Tier 3 (Bahrain, Lebanon, Oman, Saudi Arabia, and Qatar) are working towards the establishment of WGS and need advice and support for obtaining equipment, upgrading BioNumerics software and staff training on WGS [59]. However, unless food safety policies and NFCS function properly, WGS may not be the most effective tool. Besides the need to provide technical capacities, the involvement of government officials who have an understanding of how WGS works are the key factor for its implementation [87].

## 7. Food Inspection

The administration and implementation of food laws require a qualified, trained, efficient, and honest food inspection service [17]. Inspectors are expected to have an adequate understanding of the different steps of food production and the associated hazard. They should be trained to inspect food facilities and processes for compliance with hygienic and other requirements of standards and regulations, sample food during harvest, processing, storage, transport, entry-points, and make informed decisions on the suitability and compliance of food products. Skilled inspectors should be trained to be able to recognize, collect, and transmit evidence in the events of unlawful practices and violations to law.

Despite relative improvements and reform in the regulatory framework of some countries, the food control system suffers dysfunctionalities that weaken the oversight along the food chain. It is common to find different agencies (ministries and municipalities) in the same country share inspection responsibilities which create overlapping functions [32,47,75]. Deficits in skilled inspectors, different level of inspection scrutiny giving less weight to the safety of domestic products than those for exports and disparity in inspection frequencies across different areas with relative absenteeism of oversight in rural areas is also a common feature in the way food control functions in the MENA countries [32,47,81,88]. In Countries of the Eastern Mediterranean Region which include those in the MENA and Arab speaking countries, an inspection of restaurants is erratic, uneven, and covers major cities only but not rural areas [89]. In Tunisia, the private Good Agriculture Practice certification or International Organisation for Standardization (ISO) 22,000 is obligatory for all exporters to the EU, but not for domestic production [53] and this applied to other countries in the Middle East where the same standards are not always applied to control the safety of locally produced and imported food [90,91]. 

Recently, the responsible office for inspection in Morocco ONSSA received strong criticisms for overlooking the inspection and food safety monitoring activity the different nodes of the food chain, particularly the wholesale fruit and vegetable markets, and abattoirs. The office was held responsible for the unlawful practices of pesticides and the serious failure in hygiene recorded in the abattoirs (Kesrawi, 2019). Lack of coordination between stakeholders involved in food control and limited staff capacity do not commensurate with the size of the tasks entrusted to ONSSA. However, the 2018 report from Morocco’s court of auditors indicated that products for exports were subjected to stricter control of pesticide residues than those distributed in the domestic market [88]. In addition, the reports stated that the borders were not effectively controlled which led to smuggling livestock and agricultural pesticides. The misuse of pesticides and fertilizers is common in Lebanon, Kuwait, and Qatar, Egypt, Jordan, and Oman [52]. The practice often escapes the oversight of local authorities until brought up in media as a result of end-product sampling or published reports of consumer protection associations. Inspection activities in the majority of the MENA countries follow a reactive approach relying on end-products sampling, focusing on sanitation, personal hygiene, food labels instead of risk-based preventive approach which is, as far as the available information, not enforced or embedded in national standards [25,32,47,53,81,92].

In Oman and its neighboring countries, it is also reported that the number of qualified inspectors is limited and scattered in different organizations with a lack of coordination and duplication of duties and responsibilities [25]. Very few food inspectors in Oman and Kuwait have higher education degrees in sciences or related fields, while the majority have secondary school diplomas [32,81]. On the contrary, the European Commission report concluded that the inspectors in Tunisia generally had a university degree and one inspector at the time of the audit visit, had participated in several Better Training for Safer Food training sessions concerning food safety [53].

Other recent changes focused on enhancing the inspection functions of imported goods. Egypt implemented the standardization of the inspection controls to determine the safety and quality of imported grains which have a significant contribution to food security [39] and the GCC Ministers in charge of Food Safety adopted the Guide for Control on Imported Foods in the GCC region. The guide provides science-based import control systems and clearance procedures that are applied based on potential risks to consumers and emerging risks. Providing qualitative risk ranking of imported goods, the guide describes principles and regulatory requirements to be applied by the exporting country and the importing GCC countries in assuring the safety and suitability of shipments of imported food [93].

## 8. Communication, Education, and Training

The communication with stakeholders across the food chain is important in order to share and deliver information and advice on food safety issues and emerging risks. The communication activities range from providing science-based and accurate information to consumers, educational and extension services to food producers and processors, training health workers, to providing educational programs to key officials in the local authorities responsible for food control activities and surveillance. The specific training needs of food inspectors and laboratory analysts should be set as a high priority for food control activities and as means to enhance the food control expertise which will serve an essential preventive function [17].

Food safety training programs targeting the food industry and food inspectors have been in place for several years in different countries of the MENA region such as Palestine, different emirates in UAE, Jordan, and Lebanon, sometimes initiated in response to major incidences of food poisoning and scandals. Nonetheless, the impact and outreach of these programs are generally limited [92,94,95,96]. Like in other developing countries and unlike the developed countries, the government of the MENA countries is generally not involved in developing and making available training packages or guidance documents for the industry and regulatory officers. In some countries (Lebanon, Jordan, Dubai, and Egypt), these activities are to a large extent initiated and taken over by the private sector, chamber of commerce, non-governmental organizations, funded programs by international organizations and industry or trade associations which provide the food industry with the basic and advanced knowledge in food safety and HACCP. 

The contribution of these private sector’s programs to fostering consumers’ protection and ensuring safe handling of food relies on the quality of the educational and training programs, the enforcement of the law but also the programmed outreach which may be in some cases limited to a geographical area and to targeting audiences with financial capabilities. Whether these programs were effective in promoting safe practices or followed by refresher programs has not been reported in literature for the MENA countries except Lebanon where the food safety knowledge of privately and voluntarily trained food handlers was significantly higher than the untrained, yet trained food handlers did not report an appropriate level of safe practices in the kitchen. It is documented that food safety knowledge declines over time with no refreshing training [97]. Researchers’ attention and focus on this area are growing in the region as more studies were recently published on the food safety knowledge of food handlers in the food or health sector [98,99,100,101,102]. This is quite important for they revealed the importance of food safety education and identified areas where the public sector and relevant health authorities should be engaged to promote awareness in food safety. However, these remain quite a few in comparison to a large number of publications from the developed countries.

Collaborative and funded programs have also taken place to support staff capacity building. The JFDA conducted training for food technicians in the State of Palestine on food safety control and inspection procedures through the different phases of food production followed with the practical application of the latest methods of food inspection [92]. Probably one of the most important educational activities that will serve as a roadmap toward establishing an appropriate risk management system is the on-going capacity building program on food risk assessment conducted by the project “Enhancement of Regional Trade Capacities in Food Through a Harmonized Regional Conformity Assessment and Food Safety Systems in Countries of the League of Arab States” known as the Arab Food Safety for Trade Facilitation Initiative or SAFE [82]. SAFE is a regional capacity development initiative funded by the Government of Sweden’s International development agency (Sida) and implemented by the United Nations Industrial Development Organization (UNIDO) in partnership with the League of Arab States (LAS) and its specialized organizations the Arab Industrial Development and Mining Organization (AIDMO) and the Arab Organization for Agricultural Development (AOAD) [41]. 

Twelve (12) Arab food risk assessors (the first cohort of SAFE trainees) from 10 Arab countries which include Lebanon, Jordan, Egypt, Morocco, and Saudi Arabia have completed the second part of the tailored competency enhancement program in food risk assessment implemented by SAFE in collaboration with the French Agency for Food, Environmental and Occupational Health and Safety (ANSES) and Laval University (Quebec, QC, Canada). The program was designed by 10 scientists and experts to address the needs of the Arab region and to support science and risk-based regulatory decisions [82]. Moreover, Egypt established in 2004 the Egyptian Food Safety Information Center, Food Technology Research Institute, and Agricultural Research Center in endeavors to build up scientific body capable of handling and analyzing the risk identification in relation to foodborne disease data [38]. 

Regional conferences such as Oman Food Safety Conference organized by the Muscat Municipality, Ministry of Agriculture and Fisheries Wealth, Ministry Of Regional Municipal and Water Resources, and the Ministry of Commerce and Industry, and Dubai International Food Safety Conference are gaining momentum as the scientific platforms for sharing best practices, information, education, and communication among the various regional and international stakeholders. These forums play a key role in promoting food safety and initiating collaborative projects between the region and international societies. 

## 9. Conclusions

Several countries in the MENA region have made substantial efforts in improving their food safety systems and in some cases, in unifying the food control activities under one central agency. However, many challenges are still encountered due to ineffective surveillance systems, lack of communication among stakeholders, and limited, sometimes absent, food control functions along the food supply chain. MENA countries have limited capacity to enforce the law and implement food safety policies on a large scale and foster inter-communications among stakeholders. On the whole, the transition we witness in the region, although happening in slow steps, is promising but often shadowed by international organizations and institutions such as the CDC, WHO, and others.

Overall, existing information reflects that food control systems are not yet founded on scientific understanding of current risks and fall short in human resources and sometimes in skillful inspectors. At the same time, scattered reports or news on food poisoning suggest that the prevalence of foodborne diseases could be much more than what is actually reported and published. There is an extensive shortage of scientific data and information on foodborne diseases, etiological agents and causative factors to fully evaluate the food safety problems and their burdens in the region. These are instrumental for identifying common hazards, risk communications and for focusing resources and drafting policies to control the risks of high concerns. 

An effective prevention of foodborne diseases requires (1) the government officials in the MENA countries to recognize that food safety is a priority and an important public health issue, (2) enhancement in communication and collaboration among the different agencies and food chain stakeholders, (3) to focus on more research and scientific outputs to understand the local food chain dynamics and bridge the gaps with the surveillance systems, (4) to further the capacity building programs and improvements in the NFCS that are based on the risk-based approach. 

## 10. Limitations

The main limitation of this paper is the unavailability of enough data to maintain a conclusive statement on the current conditions of the food safety governance in the region. More information and knowledge about recent developments could have been obtained by organizing a focus group of experts and representatives from relevant ministries. It is important to consider direct meetings and interview with concerned stakeholders for future work on this area to allow for a better understanding and robust analysis of the national food control systems. 

In addition, Syria, Yemen, and Libya were excluded due to scarce data while also in some parts of the review, we tended to focus on selected countries depending on the availability of information pertinent to each section. 

## Figures and Tables

**Table 1:** Foodborne diseases (FBD)in the Middle East and North Africa (MENA) countries ^†^.

Country	FBD	Number of Cases	Source of Data
Lebanon *	**2003**		
	Brucellosis	193	
	Dysentery	158	[57]
	Food Poisoning	68	
	Typhoid Fever	891	
	Viral Hepatitis A	616	
	**2018**		
	Brucellosis	242	
	Dysentery	207	[66]
	Food Poisoning	459	
	Typhoid Fever	237	
	Viral Hepatitis A	899	
	**2009–2013**		
	FBD with the great majority due to salmonellosis	1747 including 4 deaths	[60]
Libya	**2001–2004**		
	Food poisoning	297 (2001)	[57]
		278 (2002)	
		129 (2003)	
		779 (2004)	
Oman	**2002**		
	Shigellosis	1158	[57]
	Amoebiasis	5440	
	Acute gastroenteritis	112,904	
	and diarrhoea		
Jordan	2002 *	(cases per one month) ^‡^	
	Salmonellosis	(271)	[57]
	Shigellosis	(1899)	
	Brucellosis	(854)	
	**2011**		
	Food poisoning	133	
	**2014**		[67]
	Food poisoning	250	[67]
Saudi Arabia	**2003**	(prevalence rate of FBD)	
	Hepatitis A	(9.55)	[57]
	Typhoid and paratyphoid	(1.83)	
	Amoebic dysentery	(10.57)	
	Salmonellosis	(10.07)	
	Shigellosis	(2.22)	
	Food poisoning outbreaks	(16)	
	**2006**		
	31 food outbreaks attributed to *Salmonella* and *S*. aureus	251	[64]
Dubai	**2011**		
	FBD	1663 (suspected)	[56]
	**2013**		
	FBD	1120 suspected (518 confirmed)	[68]
	Amoebic dysentery	214	
	Typhoid/paratyphoid	137	
	Hepatitis A	43	
	Giardiasis	20	
	Shigellosis	10	
	*Campylobacter* spp.	1	
	*E. coli*	1	
	*Campylobacter* jejuni	3	
	Bacillary dysentery	5	
	**2018**		
	Salmonellosis	200 (first half of 2018)	[69]
Morocco	**2001–2006**		
	FBD with 28% of the cases attributed to *C. perfringens*	630	[70]
	*Staphylococcus aureus*	9	
Tunisia	**2017**		
	Food poisoning	1015	[35]
	**2018**		
	Food poisoning	1855	

* The reported cases in Lebanon comprise food and waterborne diseases; ^‡^ estimated cases per one month (late summer) based on the total population of Jordan (5.3 million people in 2002) and laboratory surveys; ^†^ most of the information on foodborne illnesses are reported based on physician diagnosis and pathogen isolation from clinical specimens without corroborative evidence of consumption of contaminated food.

## References

[1] WHO (2019). Food safety. Fact Sheet.

[2] WHO (2015). WHO Estimates of the Global Burden of Foodborne Diseases. Foodborne Disease Burden Epidemiology Reference Group 2007–2015.

[3] Havelaar A.H., Kirk M.D., Torgerson P.R., Gibb H.J., Hald T., Lake R.J., Praet N., Bellinger D.C., De Silva N.R., Gargouri N. (2015). World Health Organization Global Estimates and Regional Comparisons of the Burden of Foodborne Disease in 2010. PLoS Med..

[4] Todd E.C.D., Murad S., Baydoun E., Daghir N. (2017). Foodborne Disease in the Middle East. Water, Energy & Food Sustainability in the Middle East: The Sustainability Triangle.

[5] Mirkin B. (2010). Population Levels, Trends and Policies in the Arab Region: Challenges and Opportunities.

[6] Breisinger C., Van Rheenen T., Ringler C., Nin-Pratt A., Minot N., Aragon C., Yu B., Ecker O., Zhu T. (2010). Food Security and Economic Development in the Middle East and North Africa|IFPRI: International Food Policy Research Institute.

[7] Busaidi M.A. (2017). Effective Seafood Safety, and Quality Management Systems: An Analysis of the Situation in the Sultanate of Oman. Ph.D. Thesis.

[8] El Haddad G.F., Soliman I., Mashhour A., Gaber M., Ait El Mekki A., El Hindi A., Thabet H., Thabet B., Cagatay S. (2011). A Review of the National and International Agro-Food Policies and Institutions in the Mediterranean Region.

[9] UN ESCWA (2019). Expert Group Meeting on Tracking Food Security in the Arab Region.

[10] Tropp H., Jägerskog A. (2006). Water Scarcity Challenges in the Middle East and North Africa (MENA). Human Development Occasional Papers (1992–2007).

[11] Immerzeel W., Droogers P., Terink W., Hellegers P., Bierkens M., van Beek R. (2011). Middle-East and Northern Africa Water Outlook.

[12] Faour-Klingbeil D., Todd E.C.D. (2018). The Impact of Climate Change on Raw and Untreated Wastewater Use for Agriculture, Especially in Arid Regions: A Review. Foodborne Pathog. Dis..

[13] FAO (2017). Near East and North Africa Regional Overview of Food Insecurity 2016. Sustainable Agriculture Water Management is Key to Ending Hunger and to Climate Change Adaptation.

[14] Müller S., Sievert S., Klingholz R. (2016). MENA: A Region in Crisis. The Influence of Demographic Change on Developments in the Middle East and North Africa, and What This Means for Europe.

[15] Cook S.A., Moretti L., Rudin D. (2012). Corruption and the Arab Spring. Brown J. World Aff..

[16] Arab Center Washington DC The Lebanon Uprising: Causes and Ramifications 2019. http://arabcenterdc.org/policy_analyses/the-lebanon-uprising-causes-and-ramifications/.

[17] FAO/WHO (2003). Assuring Food Safety and Quality: Guidelines for Strengthening National Food Control Systems.

[18] BAMF (2005). The Impact of Immigration on Germany’s Society. The German Contribution to the Pilot Research Study “The Impact of Immigration on Europe’s Societies“ within the Framework of the European Migration Network.

[19] FAO/WHO (2005). Food Safety Risk Analysis, PART I An Overview and Framework Manual Provisional Edition.

[20] CAC (2003). Procedural Manual.

[21] FAO Food safety and Quality: Risk Analysis 2019. http://www.fao.org/food/food-safety-quality/capacity-development/risk-analysis/en/.

[22] FAO (2006). Strengthening National Food Control Systems Guidelines to Assess Capacity Building Needs.

[23] CAC (2007). Codex Alimentarius Commission—Procedural Manuali.

[24] CAC (2013). Codex Alimentarius Commission Principles and Guidelines for National Food Control Systems-CXG 82-2013. http://www.fao.org/fao-who-codexalimentarius/codex-texts/guidelines/en/.

[25] Al-Kandari D., Jukes D.J. (2009). A situation analysis of the food control systems in Arab Gulf Cooperation Council (GCC) countries. Food Control.

[26] FAO (2004). Food Safety and International Trade in the Near East Region—Twenty-Seventh FAO Regional Conference for the Near East. http://www.fao.org/3/J1653e/J1653e.htm.

[27] EC (2016). Imports of Food of Animal Origin from Non-EU Countries. Provisions of Guarantees Equivalent to EU Requirements on Residues of Veterinary Medicines, Pesticides and Contaminants.

[28] (2018). EC Commission Regulation (EC) No 1881/2006 of 19 December 2006 Setting Maximum Levels for Certain Contaminants in Foodstuffs. Off. J. Eur. Union.

[29] UNIRAQ WHO Organizes a Food Safety and Quality Assurance Assessment Mission to Iraq. http://www.uniraq.org/index.php?option=com_k2&view=item&id=10387:who-organizes-a-food-safety-and-quality-assurance-assessment-mission-to-iraq&Itemid=605&lang=en.

[30] (2007). Bin-Fahad The Role of GSO on GCC Food Safety. Presented at Dubai International Food Safety Conference.

[31] Shehata A. (2010). Food Regulations in The Middle East: A Food Industry Perspective. Presented at Dubai International Food Safety Conference.

[32] Al-Busaidi M.A., Jukes D.J. (2015). Assessment of the food control systems in the Sultanate of Oman. Food Control.

[33] SFDA GRASF. https://grasf.sfda.gov.sa/.

[34] FAO Food Safety and Quality: Country Page. http://www.fao.org/food/food-safety-quality/gm-foods-platform/browse-information-by/country/country-page/en/?cty=TUN.

[35] Youssef A. The New Arab. https://www.alaraby.co.uk/society/2019/2/13/%D8%A7%D9%84%D8%A8%D8%B1%D9%84%D9%85%D8%A7%D9%86-%D8%A7%D9%84%D8%AA%D9%88%D9%86%D8%B3%D9%8A-%D9%8A%D8%B5%D8%A7%D8%AF%D9%82-%D8%B9%D9%84%D9%89-%D9%82%D8%A7%D9%86%D9%88%D9%86-%D8%B3%D9%84%D8%A7%D9%85%D8%A9-%D8%A7%D9%84%D8%BA%D8%B0%D8%A7%D8%A1-%D8%A7%D9%84%D8%A8%D8%B4%D8%B1%D9%8A-%D9%88%D8%A7%D9%84%D8%AD%D9%8A%D9%88%D8%A7%D9%86%D9%8A.

[36] Cortas A. (2017). The Food Safety Law in Lebanon: What Is Next?. Adv. Tech. Clin. Microbiol..

[37] Hassan T. Egypt Establishes the National Food Safety Authority—Sharkawy and Sarhan. https://sharkawylaw.com/stay-informed/1693-2/.

[38] Ibrahim N.A., Abdel-Haleem A.M.H. (2016). Food Regulations and Enforcement in Egypt. Ref. Modul. Food Sci..

[39] FAO A Renewed Focus on Food Safety in Egypt. http://www.fao.org/neareast/news/view/en/c/1201493/.

[40] AFSS Arab Food Safety Scientists (AFSS) Platform. http://afssplatform.com/.

[41] SAFE Home. https://www.english.arabsafetrade.org.

[42] The UAE’s Government Portal Food Safety—The Official Portal of the UAE Government. https://www.government.ae/en/information-and-services/health-and-fitness/food-safety-and-health-tips.

[43] El Sheikha A.F. (2015). Food Safety Issues in Saudi Arabia. Nutr. Food Technol. Open Access.

[44] FAO/WHO (2005). Regional Conference on Food Safety for Africa—Tunisian National Food Safety System.

[45] Todd E.C.D. (2017). Foodborne disease and food control in the Gulf States. Food Control.

[46] Al Batel M. Kuwait Country Report 2010. http://www.rrmiddleeast.oie.int/download/pdf/koweit%20pres%20countries/Kuwait_Food%20Safety_OIETraining_Kuwait.pdf.

[47] Faour-Klingbeil D. (2017). The Microbiological Safety of Fresh Produce in Lebanon—A Holistic “Farm-to-Fork Chain” Approach to Evaluate Food Safety, Compliance Levels and Underlying Risk Factors. Chapter 2 the Role of Inequity and Political Incoherence as Primary Risk Factors for Food Safety—A Focus on the Fresh Produce Chain. Ph.D. Thesis.

[48] Ghenghesh K.S., Belhaj K., El-Amin W.B., El-Nefathi S.E., Zalmum A. (2005). Microbiological quality of fruit juices sold in Tripoli–Libya. Food Control.

[49] El-Jardali F., Hammoud R., Kamleh R., Jurdi M. (2014). K2P Briefing Note: Protecting Consumers in Lebanon: The Need for Effective Food Safety System. https://www.aub.edu.lb/k2p/Documents/K2P%20BN%20Food%20Safety%20English.pdf.

[50] UN ESCWA (2016). Strategic Review for Food and Nutrition Security in Lebanon.

[51] Faour-Klingbeil D., Todd E.C.D. (2018). A Review on the Rising Prevalence of International Standards: Threats or Opportunities for the Agri-Food Produce Sector in Developing Countries, with a Focus on Examples from the MENA Region. Foods.

[52] Bashour I.I. (2008). Chapter 10. Pesticides, Fertilizers and Food Safety.

[53] EC (2015). Final Report of An Audit Carried out in Tunisia from 20 January 2015 to 29 January 2015 in Order to Assess the Control Systems in Place to Control Microbiological Contamination in Primary Production of Food of Non Animal Origin Intended for Export to the European Union.

[54] Apruzzese I., Song E., Bonah E., Sanidad V.S., Leekitcharoenphon P., Medardus J.J., Abdalla N., Hosseini H., Takeuchi M. (2019). Investing in Food Safety for Developing Countries: Opportunities and Challenges in Applying Whole-Genome Sequencing for Food Safety Management. Foodborne Pathog. Dis..

[55] WHO (2015). Burden of Foodborne Diseases in WHO South East Asia Region. Indd.

[56] Masudi F. Food Poisoning Cases Could Be Higher than Reported. https://gulfnews.com/uae/health/food-poisoning-cases-could-be-higher-than-reported-1.1411035.

[57] FAO/WHO (2005). FAO/WHO Regional Meeting on Food Safety for the Near East.

[58] Neeliah S.A., Goburdhun D. (2007). National Food Control Systems: A Review. Food Rev. Int..

[59] WHO (2018). Workshop on the Coordination and Capacity-Building of the PulseNet Middle East Laboratory Network.

[60] Fadlallah S., Shehab M., Cheaito K., Saleh M., Ghosn N., Ammar W., El Hajj R., Matar M. (2017). Molecular epidemiology and antimicrobial resistance of Salmonella species from clinical specimens and food Items in Lebanon. J. Infect. Dev. Ctries..

[61] Hanna N.M., Adib S.M., Daoud Z. (2009). Food-borne salmonella outbreak at a bank cafeteria: An investigation in an Arab country in transition. EMHJ-East. Mediterr. Health J..

[62] Harakeh S., Yassine H., Gharios M., Barbour E., Hajjar S., El-Fadel M., Toufeili I., Tannous R. (2005). Isolation, molecular characterization and antimicrobial resistance patterns of Salmonella and Escherichia coli isolates from meat-based fast food in Lebanon. Sci. Total Environ..

[63] Global Biodefense U.S. Navy Helps Train Iraqi Scientists on Outbreak Surveillance. https://globalbiodefense.com/2014/01/08/u-s-navy-helps-train-iraqi-scientists-on-outbreak-surveillance/.

[64] Al-Goblan A.S., Jahan S. (2010). Surveillance for foodborne illness outbreaks in Qassim, Saudi Arabia, 2006. Foodborne Pathog. Dis..

[65] Al-Mazrou Y.Y. (2004). Food poisoning in Saudi Arabia. Potential for prevention?. Saudi Med. J..

[66] MOPH Epidemiological Surveillance. https://www.moph.gov.lb/en/Pages/2/193/esu.

[67] Saraya News 250. https://www.sarayanews.com/print.php?id=279381.

[68] Khaleej Times 518 Cases of Food Borne Diseases Recorded in Dubai—News | Khaleej Times. https://www.khaleejtimes.com/nation/uae-health/518-cases-of-food-borne-diseases-recorded-in-dubai.

[69] The National Dubai Health Authorities Report 800 Cases of Food Poisoning This Year. https://www.thenational.ae/uae/health/dubai-health-authorities-report-800-cases-of-food-poisoning-this-year-1.766542.

[70] Ed-dra A., Filali F.R., Allaoui A.E., Sfendla A. (2017). Occurrence of Clostridium perfringens in sausages sold in Meknes city, Morocco. Open Vet. J..

[71] Refai M. (2002). Incidence and control of brucellosis in the Near East region. Vet. Microbiol..

[72] WHO (2004). Operational Research in Tropicaland Other Communicable Diseases.

[73] AL Bayan Foodborne Disease Monitoring Team in Dubai. https://www.albayan.ae/across-the-uae/news-and-reports/2017-01-19-1.2831589.

[74] Khaleej Times 1,663 Cases of Food-Borne Diseases in Dubai. https://www.khaleejtimes.com/lifestyle/health-fitness/1-663-cases-of-food-borne-diseases-in-dubai.

[75] Kamleh R., Jurdi M., Annous B. (2012). Management of Microbial Food Safety in Arab Countries. J. Food Prot..

[76] Essayagh T., Essayagh M., El Rhaffouli A., Khouchoua M., Essayagh S., Khattabi A. (2017). Foodborne Outbreak, Meknes, Morocco, June 2017: What We Need to Learn. J. Trop. Dis..

[77] Hibbs R.G., Weber J.T., Corwin A., Allos B.M., Abd el Rehim M.S., Sharkawy S.E., Sarn J.E., McKee K.T. (1996). Experience with the use of an investigational F(ab’)2 heptavalent botulism immune globulin of equine origin during an outbreak of type E botulism in Egypt. Clin. Infect. Dis. Off. Publ. Infect. Dis. Soc. Am..

[78] Herriman R. Food Poisoning Outbreak Strikes Another Egypt University—Outbreak News Today. http://outbreaknewstoday.com/food-poisoning-outbreak-strikes-another-egypt-university-31344/.

[79] Turis 410 Cases of Food Poisoning in Tunisia during the Current Year. https://www.turess.com/tap/115183.

[80] Salim F. Corruption Affects Tunisians’ Food Bargains for Meat and Damaged Crops. https://www.alaraby.co.uk/economy/2017/8/13/الفساد-يطاول-غذاء-التونسيين-صفقات-لحوم-ومحاصيل-تالفة.

[81] Alomirah H.F., Al-Zenki S.F., Sawaya W.N., Jabsheh F., Husain A.J., Al-Mazeedi H.M., Al-Kandari D., Jukes D. (2010). Assessment of the food control system in the State of Kuwait. Food Control.

[82] UNIDO (2017). STDF Working Group Meeting, SPS Related UNIDO Project Developments October 2016–March 2017.

[83] Cohen D., Gargouri N., Ramlawi A., Abdeen Z., Belbesi A., Al Hijawi B., Haddadin A., Sheikh Ali S., Al Shuaibi N., Bassal R. (2010). A Middle East subregional laboratory-based surveillance network on foodborne diseases established by Jordan, Israel, and the Palestinian Authority. Epidemiol. Infect..

[84] Jackson B.R., Tarr C., Strain E., Jackson K.A., Conrad A., Carleton H., Katz L.S., Stroika S., Gould L.H., Mody R.K. (2016). Implementation of Nationwide Real-time Whole-genome Sequencing to Enhance Listeriosis Outbreak Detection and Investigation. Clin. Infect. Dis. Off. Publ. Infect. Dis. Soc. Am..

[85] Kwong J.C., Mercoulia K., Tomita T., Easton M., Li H.Y., Bulach D.M., Stinear T.P., Seemann T., Howden B.P. (2016). Prospective Whole-Genome Sequencing Enhances National Surveillance of *Listeria monocytogenes*. J. Clin. Microbiol..

[86] Taboada E.N., Graham M.R., Carriço J.A., Van Domselaar G. (2017). Food Safety in the Age of Next Generation Sequencing, Bioinformatics, and Open Data Access. Front. Microbiol..

[87] Healy M.J., Tong W., Ostroff S., Eichler H., Patak A., Neuspiel M., Deluyker H., Slikker W. (2016). Regulatory bioinformatics for food and drug safety. Regul. Toxicol. Pharmacol..

[88] Kasraoui S. Court of Auditors Reveals Shocking Lack of Food Safety in Morocco. Morocco World News 2019. https://www.moroccoworldnews.com/2019/09/282504/court-of-auditors-reveals-shocking-lack-of-food-safety-in-morocco/.

[89] Tajkarimi M., Ibrahim S.A., Fraser A.M. (2013). Food safety challenges associated with traditional foods in Arabic speaking countries of the Middle East. Trends Food Sci. Technol..

[90] Hegarty V. Food Safety Challenges in the Middle East 2006. https://www.foodsafetydubai.com/resources/contentfiles/Prev-Conference/TOPIC09.pdf.

[91] Karabudak E., Bas M., Kiziltan G. (2008). Food safety in the home consumption of meat in Turkey. Food Control.

[92] JFDA (2014). Jordan Food and Drug Administration Annual Report 2014.

[93] CCAG (2015). GCC Guide for Control on Imported Foods.

[94] Emirates News Agency Food Handling Training Enhances the Safety of the Work Environment at Abu Dhabi Food Facilities. http://wam.ae/ar/details/1395302620903.

[95] PFIUPalestinian Food Industries Union. http://pfiu.org/.

[96] SHJMUN Sharjah Food Safety Program. https://portal.shjmun.gov.ae/en/pages/sfsp.aspx.

[97] McIntyre L., Vallaster L., Wilcott L., Henderson S.B., Kosatsky T. (2013). Evaluation of food safety knowledge, attitudes and self-reported hand washing practices in FOODSAFE trained and untrained food handlers in British Columbia, Canada. Food Control.

[98] Al-Nasraween M., Omari L., Al-Qutob R., Berggren V., Taha H. (2018). Food Safety Knowledge among Chicken Shawerma Food Handlers in Amman-Jordan. Arab J. Nutr. Exerc. AJNE.

[99] Alqurashi N.A., Priyadarshini A., Jaiswal A.K. (2019). Evaluating Food Safety Knowledge and Practices among Foodservice Staff in Al Madinah Hospitals, Saudi Arabia. Safety.

[100] Bou-Mitri C., Mahmoud D., El Gerges N., Abou Jaoude M. (2018). Food safety knowledge, attitudes and practices of food handlers in lebanese hospitals: A cross-sectional study. Food Control.

[101] Faour-Klingbeil D. (2015). Investigating a link of two different types of food business management to the food safety knowledge, attitudes and practices of food handlers in Beirut, Lebanon. Food Control.

[102] Fawzi M., Shama M.E. (2009). Food Safety Knowledge and Practices among Women Working in Alexandria University, Egypt. J. Egypt. Public Health Assoc..

